# P-2257. *Pseudomonas aeruginosa* and other Bacterial Infections After Lung Transplant: Risk Factors, Airway Stenosis and other Clinical Outcomes

**DOI:** 10.1093/ofid/ofae631.2410

**Published:** 2025-01-29

**Authors:** Ruben Alfonso Hernandez Acosta, Simran Gupta, Elene Chamberlin, Ella Woehl, Erin M Connolly, Audra O’Neill, Antonio Coppolino, Nirmal S Sharma, Lindsey R Baden, Ann E Woolley

**Affiliations:** Houston Methodist / MD Anderson, Houston, Texas; Brigham and Women's Hospital, Chestnut Hill, Massachusetts; Brigham and Women's Hospital, Chestnut Hill, Massachusetts; Brigham and Women's Hospital, Chestnut Hill, Massachusetts; Brigham and Women's Hospital, Chestnut Hill, Massachusetts; Brigham and Women's Hospital, Chestnut Hill, Massachusetts; Brigham and Women's Hospital, Chestnut Hill, Massachusetts; Brigham and Women's Hospital, Chestnut Hill, Massachusetts; Brigham and Women's Hospital, Chestnut Hill, Massachusetts; Brigham and Women's Hospital, Chestnut Hill, Massachusetts

## Abstract

**Background:**

*Pseudomonas aeruginosa* (PsA) and other bacterial infections after lung transplant can lead to worse patient outcomes. The aim of this study was to investigate whether isolation of microbial pathogens post-lung transplantation increased the risk of airway stenosis requiring bronchoscopic interventions.

Table 1

**Table 1.**
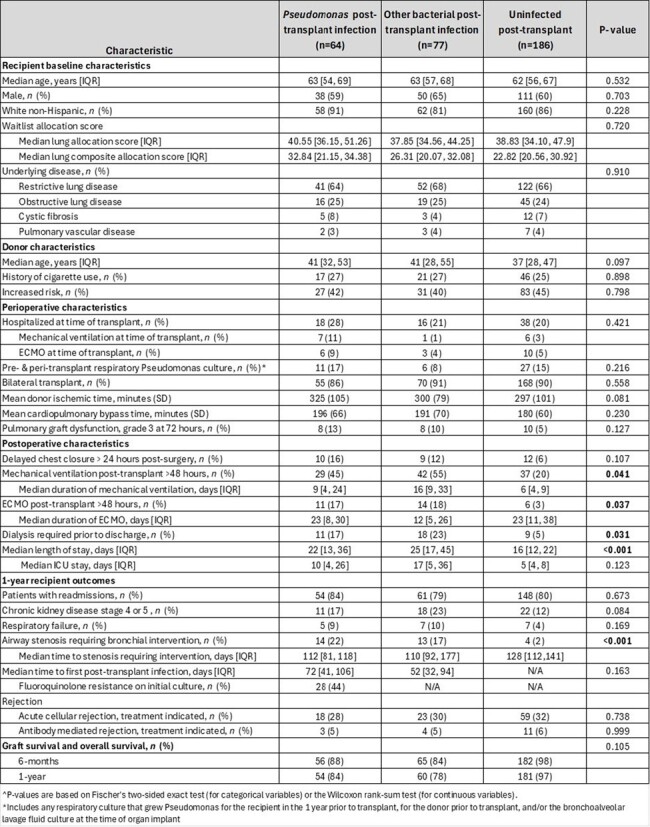
Demographic, donor/recipient characteristics, peri-operative/post-operative characteristics and outcomes.

**Methods:**

A single-center retrospective study of lung transplant recipients (LTR) between January 2017 and October 2023 was performed. Our center performs routine surveillance bronchoscopies at 1, 3, 6 and 12 months. Donor/recipient characteristics, microbiologic data, bronchoscopy findings and interventions, and 1-year outcomes were analyzed.

**Results:**

In this 6.8-year study period, 327 subjects underwent lung transplant with survival greater than 30 days. The most common indication for lung transplant was restrictive lung disease. 64 (20%) had PsA infections, 77 (23%) had non-PsA bacterial infections (infect), and 186 (57%) did not have a bacterial infection within 6-months post-transplant. Recipient and donor characteristics were similar among the cohorts. A greater proportion of the PsA and infect cohorts required dialysis and mechanical ventilation and/or ECMO for >48 hours and had a longer index hospitalization than the uninfected cohort [Table 1]. A greater proportion of the PsA and infect cohorts developed airway stenosis requiring bronchial intervention (22% vs 17% vs 2%, p=< 0.001). The median time to first infection post-transplant occurred prior to the development of stenosis (72 vs 112 days; 52 vs 110 days). 44% of LTR with PsA growth had fluoroquinolone resistance on the initial culture. Rates of rejection and 1-year survival were not statistically different among the cohorts.

**Conclusion:**

PsA and other bacterial infections within the first 6-months post lung transplantation led to a higher incidence of airway stenosis requiring bronchial intervention but was not associated with increased mortality or rejection at 1-year. The impact of treating bacterial colonization and other strategies to minimize post-transplant bacterial infections and decreasing the occurrence and time to airway stenosis needs further study.

**Disclosures:**

All Authors: No reported disclosures

